# Directly Observed Therapy and Improved Tuberculosis Treatment Outcomes in Thailand

**DOI:** 10.1371/journal.pone.0003089

**Published:** 2008-08-28

**Authors:** Amornrat Anuwatnonthakate, Pranom Limsomboon, Sriprapa Nateniyom, Wanpen Wattanaamornkiat, Sittijate Komsakorn, Saiyud Moolphate, Navarat Chiengsorn, Samroui Kaewsa-ard, Potjaman Sombat, Umaporn Siangphoe, Philip A. Mock, Jay K. Varma

**Affiliations:** 1 Thailand MOPH – U.S. CDC Collaboration, Nonthaburi, Thailand; 2 Phuket Provincial Public Health Office, Phuket, Thailand; 3 Thailand Ministry of Public Health, Nonthaburi, Thailand; 4 Office of Disease Prevention and Control 7, Ubon-ratchathani, Thailand; 5 Chiang Rai Provincial Public Health Office, Chiang Rai, Thailand; 6 Research Institute of Tuberculosis, Tokyo, Japan; 7 Bangkok Metropolitan Health Administration, Bangkok, Thailand; 8 Bamrasnaradura Institute, Nonthaburi, Thailand; 9 U.S. Centers for Disease Control and Prevention, Atlanta, Georgia, United States of America; University of Cape Town, South Africa

## Abstract

**Background:**

The World Health Organization (WHO) recommends that tuberculosis (TB) patients receive directly observed therapy (DOT). Randomized controlled trials have not consistently shown that this practice improves TB treatment success rates. In Thailand, one of 22 WHO-designated high burden TB countries, patients may have TB treatment observed by a health care worker (HCW), family member, or no one. We studied whether DOT improved TB treatment outcomes in a prospective, observational cohort.

**Methods and Findings:**

We prospectively collected epidemiologic data about TB patients treated at public and private facilities in four provinces in Thailand and the national infectious diseases hospital from 2004–2006. Public health staff recorded the type of observed therapy that patients received during the first two months of TB treatment. We limited our analysis to pulmonary TB patients never previously treated for TB and not known to have multidrug-resistant TB. We analyzed the proportion of patients still on treatment at the end of two months and with treatment success at the end of treatment according to DOT type. We used propensity score analysis to control for factors associated with DOT and treatment outcome. Of 8,031 patients eligible for analysis, 24% received HCW DOT, 59% family DOT, and 18% self-administered therapy (SAT). Smear-positive TB was diagnosed in 63%, and 21% were HIV-infected. Of patients either on treatment or that defaulted at two months, 1601/1636 (98%) patients that received HCW DOT remained on treatment at two months compared with 1096/1268 (86%) patients that received SAT (adjusted OR [aOR] 3.8; 95% confidence interval [CI] 2.4–6.0) and 3782/3987 (95%) patients that received family DOT (aOR 2.1; CI, 1.4–3.1). Of patients that had treatment success or that defaulted at the end of treatment, 1369/1477 (93%) patients that received HCW DOT completed treatment compared with 744/1074 (69%) patients that received SAT (aOR 3.3; CI, 2.4–4.5) and 3130/3529 (89%) patients that received family DOT (aOR 1.5; 1.2–1.9). The benefit of HCW DOT compared with SAT was similar, but smaller, when comparing patients with treatment success to those with death, default, or failure.

**Conclusions:**

In Thailand, two months of DOT was associated with lower odds of default during treatment. The magnitude of benefit was greater for DOT provided by a HCW compared with a family member. Thailand should consider increasing its use of HCW DOT during TB treatment.

## Introduction

Despite the widespread availability of cheap, effective treatment, tuberculosis (TB) remains a major cause of severe illness and death, with an estimated nine million new cases and two million deaths occurring annually.[1] One barrier to global TB control is the long duration of TB treatment—a minimum of six months—which frequently results in patients taking their medications erratically or not at all.[2] Non-adherence to TB medications decreases the chances of cure, increases the risk of relapse after treatment, and selects for drug-resistant TB strains.[3] Directly observing TB patients taking their anti-TB therapy, either daily or several times per week, was first piloted in the 1950s as way to insure adherence and treatment completion.[2] In 1994, based on the reported success of directly observed therapy (DOT) in increasing treatment completion rates and preventing drug resistance, the World Health Organization (WHO) adopted DOT as a principal component of its global TB control strategy.[4] Current technical manuals define DOT as direct supervision of “medication ingestion…by a treatment supporter who is acceptable and accountable to the patient and to the health system.”[5]

Although WHO and other international agencies strongly advocate DOT, controversy remains whether its benefits have been proven. Randomized controlled trials (RCT) have shown either modest or no benefit of DOT in improving TB treatment success rates, and a meta-analysis of 10 RCTs concluded that the evidence base for WHO's DOT policy is insufficient.[6] Advocates of DOT have argued that the RCTs and this meta-analysis evaluated the wrong endpoint. The effectiveness of DOT, it has been argued, should be judged by how well it prevents drug resistance, specifically to rifampin, and not by improvements in treatment success rates.[7] Others contend that the scientific literature supporting DOT has been weakened by studies involving “sloppy” DOTS; the RCTs, it has been argued, were under-powered to show an improvement in treatment success rates, because the programs studied had sub-standard TB programs.[8]

While RCTs are considered the gold standard for measuring the efficacy of a biomedical intervention, prospective observational studies are required to evaluate the effectiveness of an intervention applied to a large population in uncontrolled (i.e., real world) settings.[9] Such studies are particularly necessary for an intervention, such as DOT for TB treatment, that involves multiple components of the health system and relies predominantly on government health facilities in poor countries. Thailand is a low middle-income country with the 17^th^ largest burden of TB in the world.[1] Despite official adoption of the WHO TB control strategy in 1997, TB rates in Thailand have failed to decline, likely due to a generalized HIV epidemic and sub-optimal treatment success rates.[10] In Thailand, patients are treated in both the public and private sector, and different strategies for DOT, including no DOT, are implemented. Using data prospectively collected over two years, we evaluated the impact of different DOT strategies on treatment outcomes in a large, diverse cohort of TB patients.

## Methods

### Data Collection

In 2003, the United States Centers for Disease Control and Prevention (U.S. CDC) began collaborating with the Thailand Ministry of Public Health (MOPH), Japan's Research Institute for Tuberculosis (RIT), four provinces in Thailand (Bangkok, Ubon-Ratchathani, Phuket, Chiang Rai), and the national infectious diseases hospital (in Nonthaburi province) on the Thailand TB Active Surveillance Network, a demonstration project involving enhanced surveillance, monitoring, evaluation, and treatment of TB in Thailand.[11]

For all patients with a diagnosis of TB in the national infectious diseases hospital or any public or private facility in the four provinces, public health staff recorded standardized epidemiologic data, collected sputum specimens for microbiologic testing, and offered HIV counseling and testing. Patient data was collected prospectively from routine medical and laboratory records and entered into an electronic database. Patient outcomes were recorded through the end of TB treatment, which was usually about six months after registration.

### Patient Population

All persons registered for TB treatment were considered TB patients, consistent with WHO guidelines.[12] In this study, patients were eligible for analysis if they were registered for TB treatment between 1 October 2004 to 30 September 2006, were diagnosed with pulmonary TB, were not previously treated for TB or transferred in from a different TB program, were not known to have multidrug-resistant TB (MDR-TB), and had data recorded about their treatment observer. We classified patients with extra-pulmonary TB as ineligible, because the duration of treatment, drug regimen, and classification of outcomes, such as failure, vary depending on the location of disease.[12] We classified patients with previous TB treatment or known MDR-TB as ineligible, because such patients are known to have substantially different treatment outcomes than patients never previously treated.[13] Eligible patients were excluded from the analysis of treatment outcomes if their TB diagnosis was changed after registration, they were missing data about treatment status at two months (for the two month outcome analysis), or they were missing data about their final treatment outcome (for the end of treatment analysis). For this study, patients with an outcome of “transferred out” or patients still on treatment at the time of this analysis were considered to have missing outcome data.

### Definitions

We used standard WHO definitions to categorize patients according to previous TB treatment history, type of TB, and treatment outcome, and we classified any death which occurred during TB treatment as a TB death.[12] Consistent with WHO recommendations, sputum culture was not used to evaluate treatment outcome.[12]

In the database, public health staff recorded the type of treatment observer used during the first two months of TB treatment. Staff were instructed to classify patients as having health care worker (HCW) DOT if the patient had ingestion of anti-TB medicine observed by a HCW at least five times per week, and they were instructed to classify patients as having family DOT if the patient had a family member educated about TB treatment who was responsible for observing and recording ingestion of anti-TB medicine. Data was only recorded about the type of observer used during the first two months of treatment, because Thai national guidelines only require DOT during the period in which four drugs are administered. In actual practice, some facilities that provided DOT did so throughout treatment. The decision to allocate patients to different forms of DOT was made by individual health care providers; no data was collected about why different strategies were used in different patients.

### Data Analysis

We divided patients into three groups: HCW DOT, family DOT, or self-administered treatment (SAT). We compared the association between type of DOT received and treatment outcome at two months and at the end of TB treatment. For the two month outcome analysis, patient outcomes were divided into on treatment, died, or defaulted. We compared the proportion of patients still on treatment at two months versus those that defaulted and the proportion still on treatment versus those that either died or defaulted, according to DOT type. For the end of treatment outcome analysis, patient outcomes included successful treatment (defined as cured or completed treatment), died, defaulted, or failed. We compared the proportion of patients successfully treated versus those that died, defaulted, or failed treatment and the proportion of patients successfully treated versus those that defaulted, according to DOT type. In both the two month and end of treatment analysis, default was analyzed separately, because DOT is postulated to help reduce rates of default.[14]

In bivariate analysis, we calculated the odds ratio (OR) and 95% confidence interval (CI) for factors associated with the use of HCW, family DOT, or SAT. Statistical significance was defined as p<0.05. Because some groups of patients were more likely to receive a specific type of DOT and some factors associated with DOT use were also associated with treatment outcomes, we analyzed the association between DOT and treatment outcomes using propensity score analysis.

Propensity score analysis is used when the baseline characteristics of patients in two exposure groups (for example, those receiving HCW DOT vs. those receiving family DOT) are very different.[15] In observational studies, propensity score analyses can produce a more accurate estimate of the true association between an intervention (e.g., DOT) and an outcome (e.g., treatment success) by combining factors associated with the intervention into a composite variable, known as the propensity score, and by dividing the study population into strata that differ with respect to the likelihood of receiving the intervention, but are mostly equal with respect to other covariates.[15], [16] In this study, we first developed a multivariate logistic regression model of factors associated with DOT, constructed propensity scores based on these factors, and then divided the patient population into equally sized quintiles based on their propensity to receive DOT. We used logistic regression to calculate adjusted odds ratios for the association between treatment outcomes and DOT type controlling for the DOT propensity quintile. We used an identical approach for 12 different analyses, i.e., three exposure comparisons (HCW vs. family DOT; family DOT vs. SAT; and HCW vs. family DOT) analyzed in four different patient subsets (success vs. death, default, or failure at the end of TB treatment; success vs. default at the end of TB treatment; on treatment vs. death or default at two months; and on treatment vs. default at two months).

Because there were variables consistently associated with successful treatment or type of DOT in all 12 analysis, we included these in the calculation of propensity scores: age, gender, marital status, Thai nationality, mobility (defined as not residing for at least three of last six months in the same district), living in an urban district, chronic cough, history of injection drug use, history of being in prison, history of previous isoniazid (INH) preventive therapy, diabetes, HIV infection, having a cavity on chest radiograph, sputum culture positive for *Mycobacterium tuberculosis*, treatment with a standardized regimen (e.g., WHO Category I), and quarter of enrollment.

### Ethical Review

The protocol for this demonstration project underwent ethical review by the Thailand Ministry of Public Health and CDC and was found to be surveillance and public health program implementation, not human subjects research requiring oversight by an institutional review board.

## Results

### Patients analyzed

**Figure 1:**
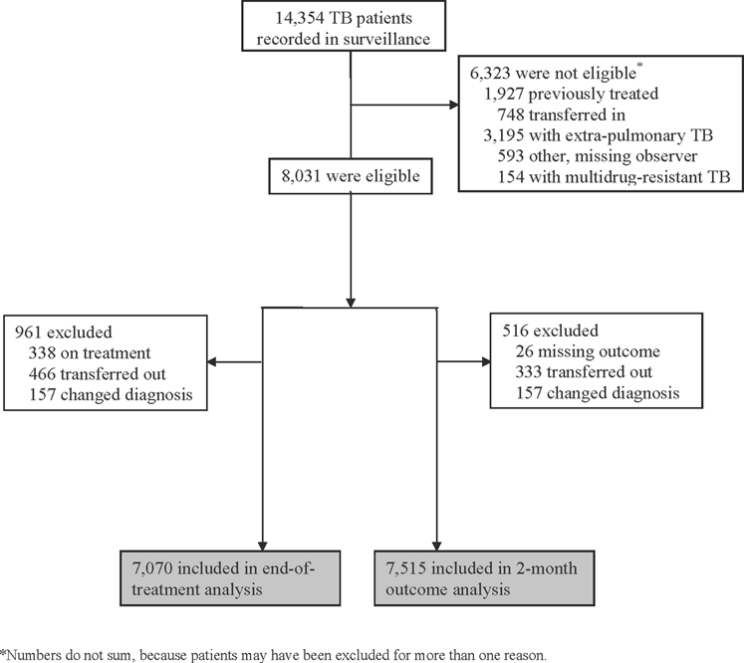
Patients included in the analysis.

Of 14,354 patients recorded in surveillance, 8,031 (56%) were eligible for the analysis.**[** **Figure 1** **]** The most common reason for non-eligibility was extra-pulmonary TB (22%). The end-of-treatment analysis included 7,070 patients, because we excluded 961 (12%) eligible patients that were recorded as still being on treatment, as transferred out, or as changed diagnosis. The two month outcome analysis included 7,515 patients, because we excluded 516 (6%) eligible patients that had an outcome recorded as transferred out, changed diagnosis, or missing.

### Characteristics of eligible patients

Of the 8,031 patients eligible for the analysis, 24% received HCW DOT, 59% family DOT, and 18% SAT.**[** **Table 1** **]** Pulmonary TB was classified as smear-positive in 5,032 (63%); overall, 47% had at least one sputum culture positive for *Mycobacterium tuberculosis*. Most patients were male, aged 15–44 years, married, and residents of a non-urban district. HIV infection was diagnosed in 1,716 (21%). Ten percent received treatment in a non-government facility; 604 (43%) of patients that received SAT were treated in a non-government facility. The standard WHO category I regimen (two months of INH, rifampin, pyrazinamide, and ethambutol, followed by four months of INH and rifampin) was prescribed to 92%; data was not available on the regimen prescribed to the other 8%. At two months, 81% of patients were still on treatment, 8% died, and 5% defaulted; the remaining patients had outcomes of transferred out (4%), or changed diagnosis (2%). At the end of TB treatment, 66% had a successful outcome (cured or completed), 23% had an unsuccessful outcome (death, default, or failure), 6% transferred out, 2% changed diagnosis, and 4% were still on treatment.

**Table 1:** Characteristics of pulmonary TB patients eligible for analysis, stratified by type of observer during first two months of TB treatment.

Characteristics	Treatment observer type			Total
	Health care worker	Family	Self-Administered	No. (%)
	No. (%)	No. (%)	No. (%)	(N = 8,031)
	(N = 1,900)	(N = 4,725	(N = 1,406)	
Type of pulmonary TB				
Smear-positive	1230 (65)	3072 (65)	730 (52)	5032 (63)
Smear-negative	662 (35)	1622 (34)	457 (32)	2741 (34)
Smear-unknown	8 (0)	31 (1)	219 (16)	258 (3)
Age				
0–14	20 (1)	99 (2)	10 (1)	129 (2)
15–44	1180 (62)	2077 (44)	948 (67)	4205 (52)
45–64	503 (27)	1435 (30)	317 (23)	2255 (28)
>65	197 (10)	1110 (24)	127 (9)	1434 (18)
Missing	0 (0)	4 (0)	4 (0)	8 (0)
Gender				
Male	1330 (70)	3091 (65)	880 (63)	5301 (66)
Female	570 (30)	1632 (35)	526 (37)	2728 (34)
Missing	0 (0)	2 (0)	0 (0)	2 (0)
Nationality				
Thai	1747 (92)	4449 (94)	1162 (83)	7358 (92)
Non-Thai	153 (8)	269 (6)	241 (17)	663 (8)
Missing	0 (0)	7 (0)	3 (0)	10 (0)
Marital status				
Married	1091 (57)	3068 (65)	543 (39)	4702 (59)
Non-Married	798 (42)	1610 (34)	632 (45)	3040 (38)
Missing	11 (1)	47 (1)	231 (16)	289 (3)
Living in an urban district				
Urban district	877 (46)	1608 (34)	823 (59)	3308 (41)
Non-urban district	1000 (53)	3001 (64)	483 (34)	4484 (56)
Missing	23 (1)	116 (2)	100 (7)	239 (3)
Mobile population*				
Non-mobile	1560 (82)	3738 (79)	467 (33)	5765 (72)
Mobile	315 (17)	836 (18)	798 (57)	1949 (24)
Missing	25 (1)	151 (3)	141 (10)	317 (4)
Ever treated with isoniazid preventive therapy (IPT)				
Previously treated with IPT	22 (1)	28 (1)	7 (1)	57 (1)
Not ever treated with IPT	1876 (99)	4688 (99)	1354 (96)	7918 (98)
Missing	2 (0)	9 (0)	45 (3)	56 (1)
Cough lasting >2 weeks at time of diagnosis				
Cough >2 weeks	1404 (74)	3258 (69)	805 (57)	5467 (68)
No cough >2 weeks	487 (26)	1389 (29)	317 (23)	2193 (27)
Missing	9 (0)	78 (2)	284 (20)	371 (5)
Ever used injection drugs				
Ever used injection drugs	80 (4)	88 (2)	36 (3)	204 (3)
Not ever used injection drugs	1769 (93)	4545 (96)	779 (55)	7093 (88)
Missing	51 (3)	92 (2)	591 (42)	734 (9)
In jail or prison				
Previously in jail or prison	80 (4)	41 (1)	7 (0)	128 (2)
Not in jail/prison	1805 (95)	4671 (99)	1006 (72)	7482 (93)
Missing	15 (0)	13 (0)	393 (28)	421 (5)
Living in migrant or refugee camp				
In camp	83 (4)	19 (0)	10 (1)	112 (1)
Not in camp	1765 (93)	4626 (98)	844 (60)	7235 (90)
Unknown	52 (3)	80 (2)	552 (39)	684 (9)
Facility that made diagnosis				
Private health facility	46 (3)	229 (5)	608 (43)	883 (11)
Government health facility	1853 (97)	4494 (95)	797 (57)	7144 (89)
Missing	1 (0)	2 (0)	1 (0)	4 (0)
Facility that provide treatment				
Private health facility	10 (1)	172 (4)	604 (43)	786 (10)
Government health facility	1889 (9)	4551 (96)	802 (57)	7242 (90)
Missing	1 (0)	2 (0)	0 (0)	3 (0)
Diabetes mellitus				
Diabetes	63 (3)	236 (5)	37 (3)	336 (4)
No diabetes	1459 (77)	3881 (82)	886 (63)	6226 (78)
Missing	378 (20)	608 (13)	483 (34)	1469 (18)
HIV status				
Positive	433 (23)	983 (21)	300 (21)	1716 (21)
Negative	1322 (70)	2834 (60)	365 (26)	4521 (56)
Missing	145 (7)	908 (19)	741 (53)	1794 (22)
Chest radiograph				
Normal	15 (1)	76 (1)	34 (2)	125 (2)
Not performed or results missing	103 (5)	550 (12)	63 (5)	716 (9)
Abnormal	1782 (94)	4099 (87)	1309 (93)	7190 (89)
Presence of a cavity	490 (28)	1164 (28)	272 (21)	1926 (27)
Sputum culture result#				
Growth of MTB	1118 (59)	2297 (49)	349 (25)	3764 (47)
No growth	400 (21)	686 (14)	127 (9)	1213 (15)
Not performed, contaminated, or grew NTM	382 (20)	1742 (37)	930 (66)	3054 (38)
Initial treatment prescribed				
CAT I (2HRZE/4HR)	1785 (94)	4454 (94)	1126 (80)	7365 (92)
Other regimens	115 (6)	271 (6)	280 (20)	666 (8)
Period of the year				
Oct.–Dec.	492 (26)	995 (21)	289 (20)	1776 (22)
Jan.–Mar.	506 (26)	1211 (26)	419 (30)	2136 (27)
Apr.–Jun.	454 (24)	1331 (28)	376 (27)	2161 (27)
Jul.–Sep.	447 (24)	1188 (25)	322 (23)	1957 (24)
Missing	1 (0)	0 (0)	0 (0)	1 (0)
Treatment outcome at the end of intensive phase				
Smear-negative	1295 (68)	2937 (62)	416 (30)	4648 (58)
Smear-positive	51 (3)	131 (3)	46 (3)	228 (3)
Died	148 (8)	412 (9)	48 (3)	608 (8)
Default	35 (2)	206 (4)	173 (12)	414 (5)
Transferred out	86 (4)	177 (4)	70 (5)	333 (4)
Change of diagnosis	29 (2)	118 (3)	10 (1)	157 (2)
On treatment, smear unknown@	256 (3)	721 (15)	640 (46)	1617 (20)
Missing	0 (0)	23 (0)	3 (0)	26 (0)
Treatment outcome				
Cured	877 (46)	1937 (41)	205 (14)	3019 (38)
Completed	492 (26)	1198 (25)	544 (39)	2234 (28)
Failure	17 (1)	74 (2)	13 (1)	104 (1)
Died	222 (12)	583 (12)	67 (5)	872 (11)
Default	108 (6)	401 (8)	332 (23)	841 (10)
Transfer out	121 (6)	245 (5)	100 (7)	466 (6)
Change of diagnosis	29 (1)	117 (2)	11 (1)	157 (2)
On treatment	34 (2)	170 (4)	134 (10)	338 (4)

* Mobile was defined as not living in the same district for at least three of the past six months.

# MTB denotes *Mycobacterium tuberculosis*, and NTM denotes non-tuberculous mycobacteria.

@ Patients who were on treatment, but had missing data about whether their sputum smears were positive or negative.

For the two month outcome analysis, the 516 patients excluded from the analysis were more likely than the 7,515 included patients to be mobile, to have known HIV status, and to have not had sputum culture performed (p<0.05, all comparisons). Other clinical and demographic characteristics were similar between the included and excluded groups. For the end of treatment analysis, the 961 excluded patients were more likely than the 7,070 included patients to have received SAT, to have not had sputum culture performed, to live in an urban district, to be mobile, and to be HIV-infected or HIV unknown (p<0.05, all comparisons).**[** **Table 2** **]**

**Table 2:** Characteristics of eligible TB patients, stratified by inclusion or exclusion from two month and end of treatment analysis.

	At Two Months		At End of Treatment	
	Excluded	Included	Excluded	Included
	No. (%)	No. (%)	No. (%)	No. (%)
	(N = 516)	(N = 7,515)	(N = 961)	(N = 7,070)
DOT				
HCW DOT	115 (22)	1788 (24)	184 (19)	1716 (24)
Family DOT	318 (62)	4407 (59)	532 (55)	4193 (59)
SAT	83 (16)	1323 (17)	245 (26)	1161 (17)
Age				
0–14	11 (2)	118 (2)	22 (2)	107 (1)
15–44	292 (57)	3913 (52)	540 (56)	3665 (52)
45–64	114 (22)	2141 (28)	235 (24)	2020 (29)
>65	98 (19)	1336 (18)	162 (17)	1272 (18)
Missing	1 (0)	7 (0)	2 (0)	6 (0)
Gender				
Male	335 (65)	4966 (66)	625 (65)	4676 (66)
Female	180 (35)	2548 (34)	335 (35)	2393 (34)
Missing	1 (0)	1 (0)	1 (0)	1 (0)
Nationality				
Thai	463 (90)	6895 (92)	876 (91)	6482 (92)
Non-Thai	52 (10)	611 (8)	84 (9)	579 (8)
Missing	1 (0)	9 (0)	1 (0)	9 (0)
Marital status				
Married	282 (55)	4420 (59)	506 (53)	4196 (59)
Non-Married	218 (42)	2822 (37)	395 (41)	2645 (37)
Missing	16 (3)	273 (4)	60 (6)	229 (3)
Living in an urban district				
Urban district	226 (44)	3082 (41)	462 (48)	2846 (40)
Non-urban district	275 (53)	4209 (56)	459 (48)	4025 (57)
Missing	15 (3)	224 (3)	40 (4)	199 (3)
Mobile population*				
Mobile	211 (41)	1738 (23)	385 (40)	1564 (22)
Non-mobile	284 (55)	5481 (73)	514 (54)	5251 (74)
Missing	21 (4)	296 (4)	62 (6)	255 (40
Ever treated with isoniazid preventive therapy (IPT)				
Previously treated with IPT	2 (0)	55 (1)	3 (0)	54 (1)
Not ever treated with IPT	512 (100)	7406 (98)	953 (99)	6965 (98)
Missing	2 (0)	54 (1)	5 (1)	51 (1)
Cough lasting >2 weeks at time of diagnosis				
Cough >2 weeks	314 (61)	5153 (68)	576 (60)	4891 (69)
No cough >2 weeks	183 (35)	2010 (27)	280 (29)	1913 (27)
Missing	19 (4)	352 (5)	105 (11)	266 (4)
Ever used injection drugs				
Ever used injection drugs	21 (4)	183 (2)	36 (4)	168 (2)
Not ever used injection drugs	462 (90)	6631 (88)	766 (80)	6372 (90)
Missing	33 (6)	701 (9)	159 (16)	575 (8)
In jail or prison				
Previously in jail or prison	12 (2)	116 (2)	23 (2)	105 (1)
Not in jail/prison	489 (95)	6993 (93)	825 (86)	6657 (94)
Missing	15 (3)	406 (5)	113 (12)	308 (5)
Diabetes mellitus				
Diabetes	15 (3)	321 (4)	40 (4)	296 (4)
No diabetes	414 (80)	5812 (77)	710 (74)	5516 (78)
Missing	87 (17)	1382 (18)	211 (22)	1258 (18)
HIV status				
Positive	152 (30)	1564 (21)	260 (27)	1456 (20)
Negative	171 (33)	1623 (22)	372 (39)	4149 (59)
Missing	193 (37)	4328 (57)	329 (34)	1465 (21)
Chest radiograph				
Abnormal	444 (86)	6746 (90)	836 (87)	6354 (90)
Normal	20 (4)	105 (1)	30 (3)	95 (1)
Not performed or results missing	52 (10)	664 (9)	95 (10)	621 (9)
Sputum culture result#				
Growth of MTB	135 (26)	3629 (48)	319 (33)	3445 (49)
No growth	88 (17)	1125 (15)	141 (15)	1072 (15)
Not performed, contaminated, or grew NTM	293 (57)	2761 (37)	501 (52)	2553 (36)
Initial treatment prescribed				
CAT I (2HRZE/4HR)	463 (90)	6902 (92)	821 (85)	6544 (93)
Other regimens	53 (10)	613 (8)	140 (15)	526 (7)
Period of the year				
Oct–Dec.	98 (19)	1678 (22)	145 (15)	1631 (23)
Jan.–Mar.	125 (24)	2011 (27)	188 (20)	1948 (28)
Apr.–Jun.	138 (27)	2023 (27)	235 (24)	1926 (27)
Jul.–Sep.	155 (30)	1802 (24)	392 (41)	1562 (22)
Missing	0 (0)	1 (0)	1 (0)	0 (0)

* Mobile was defined as not living in the same district for at least three of the past six months.

# MTB denotes *Mycobacterium tuberculosis*, and NTM denotes non-tuberculous mycobacteria.

### Outcomes at end of two months

When limited to patients who were on treatment, died, or defaulted within two months of starting TB treatment, 1,601 (90%) of 1,784 patients that received HCW DOT were still on treatment at two months, compared with 1,096 (83%) of 1,316 patients that received SAT (OR 1.8; CI, 1.4–2.2); when adjusted for propensity score, the magnitude of association decreased, and was not statistically significant (adjusted OR [aOR] 1.3; CI, 1.0–1.7).**[** **Table 3** **]** No benefit was found for patients that received family DOT vs. SAT (aOR 1.1; CI, 0.9–1.4). When comparing HCW vs. family DOT in propensity score analysis, there was no statistically significant difference in the proportion on treatment at two months versus died or defaulted.

**Table 3:** Bivariate and multivariate measures of association for successful TB treatment and health care worker observed, family member observed, and self-administered therapy.

			Treatment observer type			Analysis method	
			No. with treatment success# / No. exposed			Odds ratio	
Population Studied			(%)			(95% confidence interval)	
Outcomes	Exposures*	No. patients analyzed	Health care worker	Family member	Self-administered	Bivariate	Propensity Score Risk Adjustment
On treatment vs. death or default at two months	HCW vs. SAT	3100	1601/1784 (90%)	**—**	1096/1316 (83%)	1.8 (1.4–2.2)	1.3 (1.0–1.7)
	Family vs. SAT	5715	**—**	3782/4399 (86%)	1096/1316 (83%)	1.2 (1.0–1.5)	1.1 (0.9–1.4)
	HCW vs. Family	6183	1601/1784 (90%)	3782/4399 (86%)	**—**	1.4 (1.2–1.7)	1.1 (0.9–1.3)
On treatment vs. default at two months	HCW vs. SAT	2904	1601/1636 (98%)	**—**	1096/1268 (86%)	7.2 (4.9–10.4)	3.8 (2.4–6.0)
	Family vs. SAT	5255	**—**	3782/3987 (95%)	1096/1268 (86%)	2.9 (2.3–3.6)	2.0 (1.5–2.7)
	HCW vs. Family	5623	1601/1636 (98%)	3782/3987 (95%)	**—**	2.5 (1.7–3.6)	2.1 (1.4–3.1)
Success vs. death, default, or failure at end of TB treatment	HCW vs. SAT	2870	1369/1716 (80%)	**—**	744/1154 (64%)	2.2 (1.8–2.6)	1.6 (1.3–2.0)
	Family vs. SAT	5340	**—**	3130/4186 (75%)	744/1154 (64%)	1.6 (1.3–1.5)	1.3 (1.1–1.5)
	HCW vs. Family	5902	1369/1716 (80%)	3130/4186 (75%)	**—**	1.3 (1.2–1.5)	1.1 (0.9–1.2)
Success vs. default at end of TB treatment	HCW vs. SAT	2551	1369/1477 (93%)	**—**	744/1074 (69%)	5.6 (4.5–7.1)	3.3 (2.4–4.5)
	Family vs. SAT	4603	**—**	3130/3529 (89%)	744/1074 (69%)	3.5 (3.0–4.1)	2.0 (1.6–2.4)
	HCW vs. Family	4998	1369/1477 (93%)	3130/3529 (89%)	**—**	1.6 (1.3–2.0)	1.5 (1.2–1.9)

* HCW denotes health care worker directly observed therapy; family denotes family member directly observed therapy, and SAT denotes self-administered treatment (i.e., no directly observed therapy).

# For outcomes at two months, patients “on treatment” are considered successfully treated.

When we restricted the analysis to evaluate the impact of DOT on default at two months, HCW DOT was strongly associated with being on treatment at two months compared with SAT or family DOT in both bivariate and propensity score analysis. In patients that were either on treatment or defaulted at two months, 1,601 (98%) of 1,636 HCW DOT patients were on treatment at two months compared with 3,782 (95%) of 3,987 family DOT patients (aOR 2.1; CI, 1.4–3.1) and 1096 of 1,268 (86%) SAT patients (aOR 3.8; CI, 2.4–6.0).

### Outcomes at end of treatment

At the end of TB treatment, treatment success was associated with HCW (aOR 1.6; CI, 1.3–2.0) or family DOT (aOR 1.3; CI, 1.1–1.5) compared with SAT, among patients with treatment success, death, default, or failure. Patients who received HCW DOT were not significantly more likely to have treatment success compared with family DOT patients (aOR 1.1; CI, 0.9–1.2). When we restricted the analysis to evaluate the impact of DOT on only default at the end of TB treatment, HCW DOT was strongly associated with treatment success compared with family DOT or SAT in both bivariate and propensity score analysis. In patients that had treatment success or default at the end of treatment, 1,369 (93%) of 1,477 HCW DOT patients had treatment success compared with 3,130 (89%) of 3,529 family DOT patients (aOR 1.5; CI, 1.2–1.9) and 744 of 1,074 (69%) SAT patients (aOR 3.3; CI, 2.4–4.5).

## Discussion

In this large, prospective observational study, we found that at least two months of DOT was associated with improved TB treatment outcomes. Although observation by either a HCW or family member was beneficial, the greatest magnitude of benefit was associated with HCW DOT and the greatest impact was on treatment default rates.

This is the largest analytical study every published about the impact of DOT on TB treatment outcomes. A major strength is that it was conducted among a diverse patient population within the existing public health care system in a high burden TB country. Previous studies of DOT have been conducted at specialized centers or, when community based, involved substantially smaller or more homogenous populations.[6] In this study, over 20% of patients were HIV-infected, and patients from the private sector, urban and rural districts, and migrant (non-Thai) populations were studied. We applied rigorous statistical techniques to control for the propensity of patients to receive DOT, adjusting for the fact that patients more likely to be adherent may also be more likely to consent to DOT and that DOT may be a marker for a healthcare facility with a better performing TB treatment program.

We found that having a treatment observer was better than not having a treatment observer in reducing default. Our findings were internally consistent. Because we only recorded whether DOT was provided for the first two months of treatment, we found that the impact of DOT was greatest on default at two months. Similarly, we found a gradient of impact for DOT, with HCW DOT having a larger impact than family DOT, a finding we would have expected given that HCWs are more likely to apply DOT strictly.[17] We found less benefit when we analyzed composite endpoints of default plus death, failure, or both. We would not expect DOT to have a substantial impact on death, because the primary risk factor for death during TB treatment in Thailand is HIV, and anti-retroviral therapy is the strongest determinant of survival in HIV-associated TB.[18] We also would not expect DOT to have a substantial impact on treatment failure, because rates of failure are low in Thailand, and, when it occurs, failure is likely attributable to drug resistance.[19] Our findings are also externally consistent with previous, smaller studies conducted in Thailand and in other settings on the benefit, albeit small, of DOT.[20]–[23] DOT by family members has also been shown to produce similar outcomes as HCW DOT in one randomized trial.[24]

Our study is subject to important limitations. First, this study was conducted within the routine healthcare system. We did not independently verify that patients recorded as receiving DOT actually received DOT, nor did we measure the rigor with which DOT was applied. Misclassification would most likely have involved patients being recorded as receiving DOT but not actually receiving it, which would bias our findings to a null association.[17] Therefore, we think that this study presents a conservative estimate of the benefit to DOT. Second, because of the large number of analyses performed, we could not perfectly balance all covariates when constructing propensity score quintiles. Within a given quintile, all patients should be equally likely to receive DOT (i.e., covariates should not be statistically associated with receiving DOT within that quintile), and, across all quintiles, less than 5% of covariates should be imbalanced.[15] In our analysis, we occasionally found imbalances of greater than 5% but less than 10%. We, therefore, also conducted traditional risk factor adjusted logistic regression for all 12 analyses, and found similar direction, magnitude, statistical significance, and precision as the propensity score analysis (data not shown). Although both statistical methods are valid, they can only adjust for measured confounders; it is possible that unmeasured confounders are responsible for the association between DOT and favorable outcomes that we found. Third, we only collected data about DOT use for the first two months of TB treatment. We are not able to draw any conclusions about the impact, either positive or negative, that providing DOT for the entire duration of treatment might have. Finally, we excluded patients from the two month and end of treatment analyses because of missing data. In some TB programs, patients who transfer out but who do not have final treatment outcomes reported as part of the original cohort are considered to have defaulted. We believe that either counting these patients as defaulters or excluding them would not change our findings, because the proportion of eligible patients excluded because of being transferred out was not substantial (359 [4.5%] for the two month outcome; 466 [5.8%] for the end-of-treatment outcome).

Despite the additional public health infrastructure provided through this project, treatment outcomes remained far below international targets. Some of this can be explained by the large number of HIV-infected, sputum smear-negative, private practice, and non-Thai patients included in our analysis. Nevertheless, Thailand's overall treatment success rates remain sub-optimal. Although our findings of a strong benefit to DOT in reducing default are not completely consistent with RCTs, they are highly consistent with established public health experience, and, most important, they are generalizable to the health system within Thailand. Our study strongly suggests that Thailand's national TB program should strengthen its use of DOT and, wherever possible, use HCWs to provide it. Because a large number of patients that received SAT were treated in the private sector, efforts are also needed to bring private sector practices in line with international standards, including use of DOT.[5]
